# Methodology for examining the relationship between oil rent and crude oil production: Evidence from Cameroon

**DOI:** 10.1016/j.mex.2023.102363

**Published:** 2023-09-04

**Authors:** Marcel Rodrigue Ewodo-Amougou, Flavian Emmanuel Sapnken, Inoussah Moungnutou Mfetoum, Jean Gaston Tamba

**Affiliations:** a Laboratory of Technologies and Applied Science, PO Box 8698, IUT Douala, Douala, Cameroon; b Transports and Applied Logistics Laboratory, University Institute of Technology, University of Douala, PO Box 8698 Douala, Cameroon; c Energy Insight-Tomorrow Today, PO Box 2043 Douala, Cameroon

**Keywords:** Oil rent, Crude oil production, ARDL, Toda and Yamamoto, Cameroon, *Estimation paper and Causality Approach*

## Abstract

The relationship between oil rent and crude oil production remains unexplored in Cameroon. This study therefore aims to apply the Autoregressive Distributed Lag (ARDL) estimation technique and the Granger causality test using the Toda–Yamamoto procedure to capture the symmetric impact and causality links between oil rent and crude oil production in Cameroon. The study covers the period from 1977 to 2019, and includes crude oil prices, human development index (HDI) and corruption as other variables. The study indicates that there is a significant negative linear impact of crude oil production on oil rent and a bidirectional causality between oil rent and crude oil production. Finally, the price of crude oil, HDI and corruption are found to pass through production to influence oil rent. The results of this study will guide policy makers in managing and sustaining oil revenues for growth and prosperity.

• The paper examines the linear impact of crude oil production on oil rent and the causal links between crude oil production and oil rent by incorporating crude oil prices, HDI and corruption.
• Bidirectional causality between oil rent and crude oil production.
• Convergence of crude oil price, HDI and corruption to crude oil production to influence oil rent.

## Graphical abstract

**Figure:**
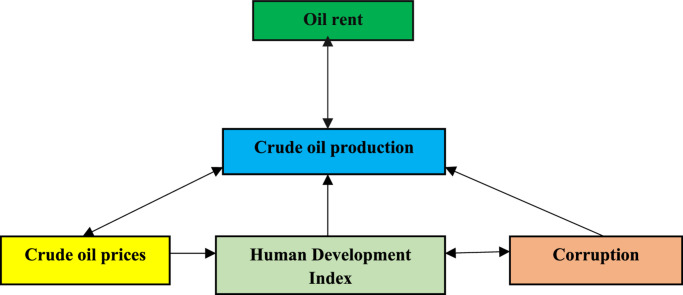

Specifications table

**Table:** 

Subject area:	Energy
More specific subject area:	*Oil resources policy, econometrics*
Name of your method:	*Estimation paper and Causality Approach*
Name and reference of original method:	*Pesaran, M.H., Shin, Y., Smith, R.J., 2001. Bounds testing approaches to the analysis of level relationships. J. Appl. Econ. 16, 289-326.*https://doi.org/10.1002/jae.616 *Toda, H.Y., Yamamoto, T., 1995. Statistical inference in vector autoregressions with possibly integrated processes. Journal of Econometrics 66, 225-250.*https://doi.org/10.1016/0304-4076(94)01616-8
Resource availability:	WDI, 2022. World Development Indicators | DataBank [WWW Document]. URL https://databank.worldbank.org/source/world-development-indicators# (accessed 9.1.22) SNH, 2021b. Key data [WWW Document]. URL https://www.snh.cm/index.php/en/hydrocarbons-in-cameroon/key-data (accessed 9.1.22) NUDP, 2022. Human Development Reports. United Nations. Gankou, J.-M., Bendoma, M., Sow, M.N., 2016. The Institutional Environment and the Link between Capital Flows and Capital Flight in Cameroon. African Development Review 28, 65-87. https://doi.org/10.1111/1467-8268.12182

## Data and methodology of the ARDL model for Co-integration

The annual time series data used in this research covers the period from 1977 to 2019. The year 1977 marks the start of crude oil exploitation in Cameroon and the year 2019 was chosen as the end of the study period because of the official data structure in Cameroon which is limited to this date.

Our analysis is based on the existence in the literature of works dealing with the relationship between resource rents (oil rents only) and crude oil production in a geopolitical risk context, mainly those of [1] on the link between crude oil production and resource rents (oil rents of 8 Persian Gulf countries). Other existing studies have mentioned the link between crude oil production and oil rent, including [2], those of [3] and those of [4]. In this study, we also include the price of crude oil as an important control variable. A higher crude oil price allows countries to earn more oil rent [5] and shocks to oil rents are generally driven in part by crude oil prices [4]. Finally, our analysis also includes two other major determinants of oil rent, namely the HDI and corruption. Human development is seen as a remedy to be used to offset the negative effect of oil revenues on financial development [6] and corruption has been shown to be an important factor in oil revenues through the establishment of the EITI [7]. In light of the existing work, the general factors of oil rent (RE) can be defined by the following elements: crude oil production (PR), crude oil price (PX), HDI and corruption (CO). This research proposes the following empirical model:

(1) $$RE_{t}=f\left(PR_{t,\;}\;PX_{t,\;}\;HDI_{t,\;}\;CO_{t}\right)\;$$

All variables are transformed into natural logarithm following [8] in Eq. (1) except for the corruption variable (CO) to obtain consistent and reliable empirical results [9]. The natural logarithm thus allows smoothing the different variables and making interpretations in the form of elasticity. The empirical model between oil rent and all other variables is stated as follows:

(2) $$lnRE_{t}=\;\alpha_{0}+\;\alpha_{1}lnPR_{t}+\;\alpha_{2}lnPX_{t}+\;\alpha_{3}lnHDI_{t}+\;\alpha_{4}CO_{t}\;$$

In Eq. (2), $lnRE_{t}$, $lnPR_{t}$, $lnPX_{t}$, $lnHDI_{t}$ represent the natural logarithm of oil rent, crude production, crude price and HDI respectively. $CO_{t}$ represents corruption, $\alpha_{0}$ the constant and $\varepsilon_{t}$ the error term.

Before running the time series model, it is imperative to check the nature of the data series [10]**.** Therefore**,** this study examined the stationarity of all variables using the augmented Dickey–Fuller stationarity test [11] and the Zivot–Andrews [12]**.** These tests are used in this study for several reasons. The first reason is that the ARDL model generally suffers from an error autocorrelation problem due to the presence of the lagged endogenous variable among the explanatory variables of the model. The ADF test is therefore indicated for this purpose. Several authors have used the ADF test in the literature to confirm the stationarity of the series. We can cite the work of Sharif et al. [13] and Rehman et al. [14] and Yang et al. [15]. The second reason is that the endogenous variable suffers from a rather large volatility problem [[16], [17], [18], [19]]. This is the reason why this study takes into account the Zivot–Andrews (AZ) test which is very suitable for series that are victims of regime shifts. This test has been used in the literature by Mallick et al. [20] and Zafar et al**.** [21]**.**

In order to examine the existing relationship between oil rent and crude oil production in Cameroon, the study establishes an ARDL model, uses the Co-integration test of Pesaran et al. [22] reported by Narayan [23] and finally it considers Granger causality in the Toda-Yamamoto sense. This scheme was chosen for several reasons. Firstly, the issue of endogeneity is incorporated into the ARDL model by including lags for the dependent and independent variables; it is therefore an efficient technique for estimating short and long term Co-integration relationships [24]. Secondly, the ARDL model does not require that all the variables in the model integrate at the same order unlike Johansen's Co-integration; it can therefore be applied when the variables are integrated of order one, order zero or even a combination of both [22]. Thirdly, the ARDL model applies to a small sample size [25,26]. Fourth, the ARDL model represents the data creation process typically used in specific modelling frameworks because of its ability to handle a large number of delays [22]. Finally, even when the explanatory variables are endogenous, the ARDL model provides correct long-run model estimates and valid t-statistics [22]. The test ARDL model used in this study is the following:

(3) $$\begin{array}{cc} \Delta \;\ln RE_{t} & =\alpha_{0}+\sum_{i=1}^{t}\beta_{i}\;\Delta \;\ln RE_{t-i}+\sum_{i=0}^{t}\rho_{i}\;\Delta \;\ln PR_{t-i}+\sum_{i=0}^{t}\varphi_{i}\;\Delta \;\ln PX_{t-i}+\;\sum_{i=0}^{t}\omega_{i}\;\Delta \;\ln HDI_{t-i}\\ & \;+\sum_{i=0}^{t}\Phi_{i}\Delta CO_{t-i}\;+\lambda_{1}\;\ln RE_{t-1}+\lambda_{2}\;\ln PR_{t-1}\;+\lambda_{3}\;\ln PX_{t-1}+\lambda_{4}\;\ln HDI_{t-1}+\lambda_{5}CO_{t-1}+\varepsilon_{t} \end{array}$$

In Eq. (3),$\;lnRE_{t}$, $lnPR_{t}$, $lnPX_{t}$, $lnHDI_{t}$ represent the logarithmic values of oil rent, crude oil production, crude oil price, human development index (HDI), and CO represents corruption, respectively; αₒ the constant; $\beta_{i},\;\rho_{i},\;\varphi_{i},\;\omega_{i}$ and $\phi_{i}$ represent the short-run effects (elasticities); *λ*_1_...*λ*_5_ *represent the long-term* effects (elasticities) of the model and $\varepsilon_{t}$ the error term (white noise). Δ is the first difference operator and t is the maximum number of lags in the model based on the Schwarz information criterion (SIC). There are other information criteria such as the AIC criterion and the HQ criterion. The criterion is chosen based on the lowest value of the shift length selection criteria. The work of Khan et al. [27] allowed us to make the choice of the criterion for this study.

In order to study the long-run Co-integration relationship between variables, the literature provides several econometric techniques. For example, the Engle and Granger test [28]; those of Johansen [29,30] and Pesaran et al**.** [22]. The Engle and Granger Co-integration test [28] is applied to check the Co-integration only between two integrated series of the same order. It is therefore adapted to the bivariate case and is therefore less efficient for multivariate cases. To correct this, the Johansen Co-integration test [29,30] was developed and allowed to check the co-integration on more than two series instead. It was developed for multivariate cases. However, although the Johansen test based on vector error correction modelling (VECM) is a remedy to the limitations of the Engle and Granger test, it also requires that all series or variables are integrated of the same order. This is not always the case in practice. Therefore, when there are several integrated variables of different orders I(0) and I(1), reference can be made to the Co-integration test of Pesaran et al**.** [22]. This study favours the method of Pesaran et al. [22] for several reasons. The first reason is that our series are integrated at different orders I(0) and I(1). The second reason is that it allows us to combine short-run dynamics and long-run effects using the error correction model as a basis.

Then, to judge the Co-integration or long-run relationship between the endogenous and exogenous variables, the Wald restriction test is used. The value of the F-test is taken by applying the diagnostic of the Wald restriction test coefficient on the parameters of the long-run variable. The assumptions for the Co-integration are:

$H_{0}\;:\lambda_{1}=\lambda_{2}=\lambda_{3}=\lambda_{4}=\lambda_{5}$ (There is no Co-integration) and,

$H_{1}\;:\lambda_{1}\neq \lambda_{2}\neq \lambda_{3}\neq \lambda_{4}\neq \lambda_{5}$ (There is Co-integration)

If the calculated F-stat value is greater than the upper bound value, then the null hypothesis will be rejected, thus concluding the existence of a Co-integration. This means that the long term relationship exists between the independent and dependent variables. When the value of the F-stat is less than the lower bound value, then the null hypothesis is not rejected; this indicates that the dependent and independent variables do not have a long term relationship because they do not show Co-integration. Finally, the result will be considered inconclusive if the F-stat is between the lower and upper bounds.

The Co-integration approach allows us to perform a Fisher test on the selected ARDL equation with appropriate lag lengths. In this study, we used the bounds reported by Narayan [23] for the small sample size of 30≤n≤80 to support the decision on the Co-integration estimates. The next step is to estimate the long and short term results and perform diagnostic tests to ensure the stability of the model. The error correction model can be formulated as follows:

(4) $$\begin{array}{cc} \Delta \;lnRE_{t} & =\beta_{0}+\sum_{i=1}^{t}\beta_{i}\;\Delta \;lnRE_{t-i}+\sum_{i=0}^{t}\rho_{i}\;\Delta \;lnPR_{t-i}+\sum_{i=0}^{t}\varphi_{i}\;\Delta \;lnPX_{t-i}+\;\sum_{i=0}^{t}\omega_{i}\;\Delta \;lnHDI_{t-i}\\ & \;+\sum_{i=0}^{t}\Phi_{i}\;\Delta CO_{t-i}\;+\theta_{i}ECM_{t-1}+\varepsilon_{t} \end{array}$$

In Eq. (4), $\theta_{i}\;$is the coefficient of the error correction term - $ECM_{t-1}\;$which measures the speed of short-term adjustment towards the long-term equilibrium path of the estimated ARDL model. The coefficient of the error correction term should be a negative and significant sign. The verification of the model diagnosis is very important in that it allows the model to be validated. Thus, to check for serial independence, the Breusch–Godfrey LM test of serial correlation and the normality test are used. The ARCH test is also used to check for heteroscedasticity in the model and the Ramsey Reset test is applied to check for misspecification in the model. Recursive CUSUM and CUSUMSQ [31] are used to detect whether an autoregressive structure is creeping into the model. These tests are used to ensure the stability of the model parameters.

The literature has mainly used vector autoregression (VAR) and error correction model (ECM) techniques to confirm Granger causality. We use the VAR model if the selected time series variables do not exhibit Co-integration characteristics. The ECM model is used if the variables are known to be Co-integrated. This means that we have to check the order of integration of the selected variables by first determining whether they are integrated, Co-integrated or stationary. Toda and Yamamoto [32] claimed that nuisance parameters in a fixed sample have a significant impact on the pre-tests of Co-integration in the Johansen types of ECM model. Therefore, the understanding of causality could be affected by the pre-test bias [33,34]. To address the problem, Toda and Yamamoto [32] suggested the augmented VAR model to examine causality, which can be applied at any arbitrary level of integration order. Zapata and Rambaldi [35] argued that when there is uncertainty as to whether the selected variables are I(0) or I(1), then the best model is to apply the Toda and Yamamato causality test because it has higher power in temperate panel sizes and does not need to worry about the order of variables. Therefore, this study adopts the Granger causality test in the sense of Toda and Yamamoto to analyse the influences that might exist between the different sub-study variables.

In this study, since the variables are integrated at different orders I(0) and I(1), the traditional Granger causality test becomes ineffective. In this case, we use the Granger causality test in the sense of Toda and Yamamoto [32] which is based on a modified Wald test (MWALD). Wolde–Rufael [13,14] and Zapata and Rambaldi [35] reported that the Modified Wald Test (MWALD) avoids the problems associated with the ordinary Granger causality test by ignoring any possible non-stationarity or co-integration between the series. The main advantage of this approach, compared to a simple unrestricted vector autoregressive model (VAR), is the applicability of this method even if the VAR is stationary or integrated of random order. This technique allows testing for causality in the Granger sense by avoiding undesirable results related to the power and size properties of unit root and Co-integration tests [35]. The approach of Toda and Yamamoto (1995) fits a VAR model to the levels of the variables, thus minimising the risks associated with a possible incorrect identification of the order of integration of the series [36,37]. The procedure of the Granger causality test proposed by Toda–Yamamoto (1995) is as follows:

i. Find the maximum integration order of the series under study ($d_{max}$) using conventional stationarity tests.
ii. Determine the optimal lag of the VAR in sub-study level (*k)* or autoregressive polynomial (AR) using the SIC information criteria;
iii. Estimate a level-augmented VAR of order ($k+\;d_{max}$).
iv. Check the robustness of the VAR$\;(k+\;d_{max})$ using available diagnostic measures.
v. Finally, a Wald test is performed on q initial parameters, which provides an asymptotic chi-squared distribution with q degrees of freedom (For more information, see [32,33])

For the estimation of the augmented level VAR, the stationarity conditions of the series will define the number of lags to be added to the VAR. In fact, for stationary series in level, no lag is added to the VAR (standard test procedure). On the other hand, for I(1) series, a lag will be added to the VAR, and so on. The models below are therefore expressed and translate the Granger causality relations in the Toda-Yamamoto sense.

**Model 1:** Oil rent (RE) and crude oil production (PR)

(5) $$\begin{array}{cc} lnRE_{t} & =\;\alpha_{0}+\;\sum_{i=1}^{k}\beta_{1i}lnRE_{t-i}+\sum_{j=k+1}^{d_{max}}\;\beta_{2j}lnRE_{t-j}+\sum_{i=1}^{k}\lambda_{1i}lnPR_{t-i}+\sum_{j=k+1}^{d_{max}}\lambda_{2j}lnPR_{t-j}\\ & \;+\sum_{i=1}^{k}\Phi_{1i}lnPX_{t-i}+\sum_{j=k+1}^{d_{max}}\Phi_{2j}lnPX_{t-j}+\sum_{i=1}^{k}\varphi_{1i}lnHDI_{t-i}+\sum_{j=k+1}^{d_{max}}\varphi_{2j}lnHDI_{t-j}+\sum_{i=1}^{k}\rho_{1i}CO_{t-i}\;\\ & \;+\sum_{j=k+1}^{d_{max}}\rho_{2j}CO_{t-j}+\;\varepsilon_{1t} \end{array}$$

**Model 2:** Crude oil production (PR) and oil rent (RE)

(6) $$\begin{array}{cc} lnPR_{t} & =\;\alpha_{1}+\;\sum_{i=1}^{k}\beta_{1i}lnRE_{t-i}+\;\sum_{j=k+1}^{d_{max}}\beta_{2j}lnRE_{t-j}+\sum_{i=1}^{k}\lambda_{1i}lnPR_{t-i}+\sum_{j=k+1}^{d_{max}}\lambda_{2j}lnPR_{t-j}\\ & \;+\sum_{i=1}^{k}\Phi_{1i}lnPX_{t-i}+\sum_{j=k+1}^{d_{max}}\Phi_{2j}lnPX_{t-j}+\;\sum_{i=1}^{k}\varphi_{1i}lnHDI_{t-i}+\sum_{j=k+1}^{d_{max}}\varphi_{2j}lnHDI_{t-j}+\sum_{i=1}^{k}\rho_{1i}CO_{t-i}\\ & \;+\sum_{j=k+1}^{d_{max}}\rho_{2j}CO_{t-j}+\;\varepsilon_{2t} \end{array}$$

Oil rent causes crude oil production if $\lambda_{1i}\neq 0$, ∀i=1,2,…,k in Eq. (5). Similarly, crude oil production causes oil rent if$\;\beta_{1i}\neq 0$, ∀i=1,2,…,k in Eq. (6). There is a bidirectional causality between oil rent and crude oil production if $\lambda_{1i}\neq 0\;$and $\beta_{1i}\neq 0$, ∀i=1,2,…,k in Eqs. (5 and 6) respectively. Finally, there is no causality between oil rent and crude oil production if $\lambda_{1i}=\beta_{1i}=0$, ∀i=1,2,…,k in Eqs. (5 and 6) respectively. The same causal inference can be made with the other models.

## Empirical results

### Descriptive statistics

Table 1 presents the simple descriptive characteristics for the selected variables from 1977 to 2019. Oil rent is the most volatile variable among the selected variables in this study in terms of standard deviation [38]. Apart from oil rent, crude oil production and corruption, the variables crude oil price and HDI are normally distributed in the model with respect to the Jarque–Bera [38]. In this case, heteroscedastic modelling will be preferred in the presence of ARCH effects, which will allow the variance of the errors to be taken into account in an autoregressive manner conditional on past information.

**Table 1:** Descriptive statistics.

	ln RE	ln PR	ln PX	ln HDI	CO
Average	6.261172	3.507095	3.879895	-0.729305	0.581395
Median	6.372261	3.572346	3.975186	-0.713350	1.000000
Maximum	7.708990	4.182661	4.939497	-0.579818	1.000000
Minimum	0.862890	0.770108	2.721295	-0.911303	0.000000
Standard deviation	1.148782	0.596480	0.629242	0.108411	0.499169
Asymmetry coefficient	-2.594122	-2.590393	-0.240730	-0.166043	-0.329983
Flattening coefficient	12.77965	12.19824	1.900524	1.627442	1.108889
Jarque-Bera	219.5855	199.6781	2.581168	3.572937	7.187910
Probability	0.000000	0.000000	0.275110	0.167551	0.027489
Sum	269.2304	150.8051	166.8355	-31.36010	25.00000
Sum of squares	55.42740	14.94311	16.62973	0.493620	10.46512
Observations	43	43	43	43	43

### Results of the stationarity tests

The stationarity properties of the variables are first investigated before examining the linear impact and causality links between oil rent and crude oil production in Cameroon. The study uses the Augmented Dickey-Fuller (ADF) test, the Andrews and Zivot (AZ) test and the results of the unit tests of the roots of the variables in levels and at first differences are contained in Table 2. These show that all oil rent is integrated of order 0 and crude oil production is integrated of order 1. The same is true for the other variables, crude oil price, HDI and corruption. This allows the study to test the long-run impact of crude oil production on oil rent in Cameroon, as well as the other variables.

**Table 2:** Stationarity of variables.

Variables	Level			First Difference			Decision
	ADF	AZ	Breakdown date/AZ	ADF	AZ	Breakdown date/ AZ	
ln RE	-7.10⁎⁎⁎ (0.00)	-8.00⁎⁎⁎ (0.01)	2004	–	–	–	I(0)
ln PR	-0.50 (0.49)	-9.84⁎⁎⁎ (0.01)	1987	-6.95⁎⁎⁎ (0.00)	–	–	I(1)
ln PX	-0.31 (0.56)	-2.50 (0.90)	1983	-5.30⁎⁎⁎ (0.00)	-6.51⁎⁎⁎ (0.01)	1985	I(1)
ln HDI	-3.17 (0.10)	-3.96 (0.16)	2007	-4.56⁎⁎⁎ (0.00)	-7.65⁎⁎⁎ (0.01)	1982	I(1)
CO	-1.87 (0.34)	-6.33⁎⁎⁎ (0.01)	1992	-6.18⁎⁎⁎ (0.00)	–	–	I(1)

**Note.** ln RE, ln PR, ln PX, and ln HDI are the logarithmic forms of oil rent, crude oil production, crude oil price, and human development index; while CO is Corruption index

⁎⁎⁎ Significance at 1%; (.): Probability

### Test results for Co-integration

The F-statistic for the study period obtained from the ARDL-related Co-integration test shows evidence of Co-integration as it is above the critical values proposed by Pesaran et al. [22] and modified by Narayan [23] as shown in Table 3. Since the equations used in the study show evidence of Co-integration between the variables, the study estimates the model using the ARDL bounds testing approach. The Schwarz Information Criterion (SIC) was used to select the optimal lag length in the study. The optimal model chosen for the study period is ARDL(1,0,0,3,0). The long and short term results of the selected model are presented in Table 4 and Table 5 for the period 1977 to 2019.

**Table 3:** Boundary Co-integration test.

Statistical test	Value	Significant.	I(0)	I(1)
F-statistic	6.271403	10%	2.66	3.838
		5%	3.202	4.544
		1%	4.428	6.25

**Table 4:** Long-term and short-term results.

Variables	Coefficient	Std. Error	t-Statistic	Prob.
**Long-run**				
ln PR	-0.819084	0.415508	-1.971283	0.0477⁎⁎
ln PX	0.576562	0.275079	2.132341	0.0410⁎⁎
ln HDI	2.364483	1.914709	2.383904	0.0434⁎⁎
CO	-0.113809	0.353553	1.255281	0.6888
**Short-run**				
D(ln RE(-1))	-0.688289	0.143247	-4.804924	0.0000⁎⁎⁎
D(ln PR)	-0.934586	0.895952	-1.043120	0.3058
D(ln PX)	0.458183	0.171737	2.667933	0.0125⁎⁎
D(ln HDI)	-3.287820	1.548623	-2.123061	0.4418
D(ln HDI(-1))	0.561716	1.123217	0.500096	0.6218
D(ln HDI(-2))	-0.388122	0.995641	-0.389822	0.7003
D(ln HDI(-3))	-0.857245	3.763209	-0.227796	0.8218
D(CO)	-0.158990	0.211238	-0.752658	0.4579
ECT(-1)	-0.688289	0.121559	-5.662178	0.0000⁎⁎⁎

⁎⁎ Significance at 5%.

⁎⁎⁎ Significance at 1%

**Table 5:** Model robustness tests.

Robustness tests	Prob	Results
Ramsey Reset test	0.97	The model is perfectly specified
Breusch-Godfrey Serial Correlation LM Test	0.81	No serial correlation
Jarque-Bera (Normality Test)	0.62	The residues are normally distributed
ARCH Test (Heteroscedasticity Test)	0.50	No evidence of heteroscedasticity
CUSUM		Stable
CUSUMSQ		Stable

Table 4 presents the long-run and short-run results**.** In the long run, the linear impact of crude oil production on oil rent is negative and significant at the 5% level. Thus, a 1% increase in crude oil production leads to a 0.81% decrease in oil rent. However, this result refers to the different shocks suffered by the oil rent and whose cause is generally the changes in production [4]**.** Moreover, the linear impacts of the crude oil price and HDI are positive and significant at the 5% level. Thus, a 1% increase in the price of crude oil and in HDI leads to an increase of 0.57% and 2.36% in oil rent respectively. These results show that the price of crude oil stimulates the oil rent [5] and that human development plays a catalytic role on oil rent [6]. Corruption remains insignificant. In the short term, crude oil production has a negative and insignificant effect on oil revenues. The same is true for HDI and corruption. The price of crude oil is the only variable that has a positive and significant impact on the oil rent. Thus, a 1% increase in the price of crude oil leads to a 0.45% increase in oil rent. Table 5 presents the results of the diagnostic and stability tests after estimation on the model. The model passed all diagnostic tests, as well as the stability tests, which proves that the results are reliable.

**Fig. 1:**
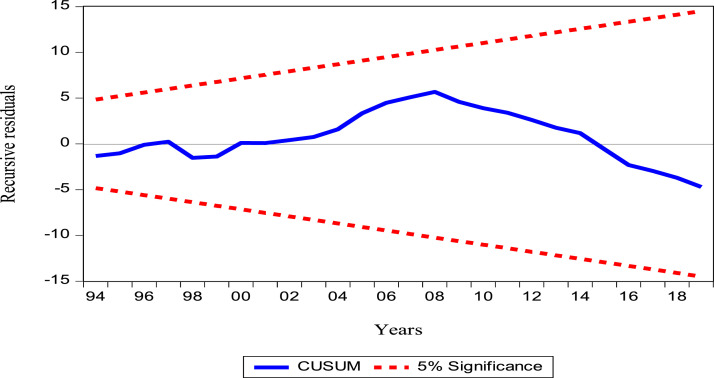
Plots cumulative sum of recursive residuals (CUSUM).

**Fig. 2:**
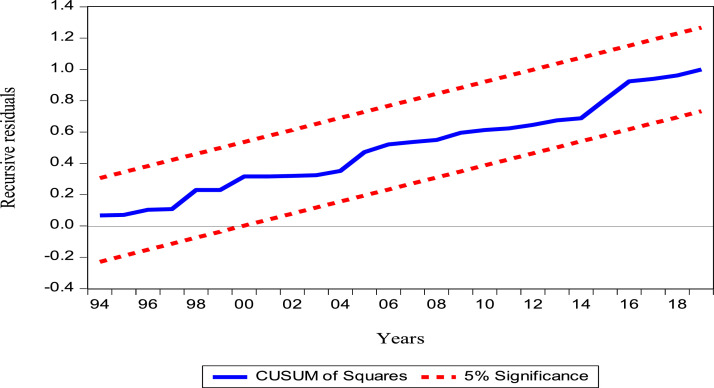
Plots cumulative sum of squares recursive residuals (CUSUMSQ).

The graphical representation of CUSUM and CUSUM SQUARED are given in Figs. 1 and 2. respectively. According to the guideline, if the plots remain within the critical limit of 5%, it means that the model is stable and consistent. Our model plots show that the CUSUM and CUSUM SQUARED are within limits and over time for the Cameroon data.

After estimating the long-term and short-term results, we proceeded to the causality test. According to the estimation of the Granger causality test in the Toda-Yamamoto sense, the results are contained in Table 6.

**Table 6:** Toda-Yamamoto causality.

Dependent variables	Explanatory variables					Decisions
	ln RE	ln PR	ln PX	ln HDI	CO	
ln RE		5.74**	0.97	2.52	0.60	PR → RE
ln PR	27.62***		7.01***	3.04*	9.04***	RE → PR, PX → PR HDI → PR, CO → PR
ln PX	1.11	3.78*		0.04	0.15	PR → PX
ln HDI	1.81	0.15	12.03***		2.81*	PX→HDI, CO→ HDI
CO	0.01	1.09	0.59	2.88*		HDI → CO

Table 6 above shows that production influences the oil rent [4] and oil rent influences production [1]**.** If crude oil production does not generate significant profits, it is likely to stop. On the other hand, if the production of crude oil brings enough profits, then crude oil will continue to be produced. Thus, there is a clear impact of oil rent on crude oil production, so that the profits from the sale of oil even determine the existence of crude oil production. Conversely, production in a favourable environment (e.g. high crude oil prices and absence of corruption) allows crude oil producing countries to reap considerable gains. Otherwise, these countries will see their earnings decrease exponentially. Oil revenues are thus conditioned by production.

In addition, the price of crude oil influences the production of crude oil [39,1] and production influences the price of crude oil [40,41]**.** If the price remains high in a context where the oil fields are located either in a hostile environment or in difficult exploitation conditions, then the oil producing multinationals will be very cautious. Otherwise, multinationals will produce large stocks of crude oil. It is therefore clear that crude oil production depends on the price of crude oil. In the opposite direction, and according to the principle of supply and demand in a market, if the supply of crude oil is greater than the demand for crude oil, then there will be an abundance of crude oil in the market leading to a deterioration of the price of crude oil. Conversely, if the supply of crude oil is less than the demand for crude oil in the market, then there will be a shortage of crude oil in the market, causing crude oil to rise. The price thus depends on the interplay between the quantity of oil offered and the quantity of oil demanded.

Also, HDI through health, education and training allows for a skilled workforce to improve productivity. Thus, improved productivity can contribute to better production. Otherwise, it is production that is poor. This result is in line with the work of Châteauneuf-Malclès [42]**.**

Finally, a unidirectional causality from corruption to crude oil production**.** Corruption can reinforce sub-optimal oil production, i.e. production below its potential [43]. Thus, it is understood that the presence of corruption in oil producing countries can lead to crude oil production at levels below the true potential of individual wells. Moreover, increases in corruption are associated with significantly lower levels of oil production in the sample [44]**.**

## Discussion of the results

Crude production has a significant negative impact on oil rent in the long run. This result could mean that Cameroon is in the grip of the oil resource curse. However, not continuing to increase crude production is not an optimal solution as Cameroon intends to increase its investment in crude production to boost its oil rent in order to develop its economy [45]. Appropriate policies must therefore be used to reverse this trend. This result is consistent with the work of [4] on the effects of oil rent shocks on economic growth. Indeed, the observed changes in production are responsible for the observed shocks in the oil rent.

Also, there is a bidirectional causality between oil rent and crude production. Indeed, the production of crude has perverse effects on the oil rent. This being the case, the oil rent could therefore compromise the exploitation of crude. For its emergence, Cameroon relies on oil revenues which will not only balance its budget but also contribute to its development [46]. Reliable mechanisms must therefore be used to meet this challenge. It should be noted that the work of [4] and those of [1] respectively indicate the possibility that production influences oil rent and that oil rent influences crude production. This reassures the results of this study. In addition, the econometric results found.

Crude oil is seen as a curse for Africa, particularly for the Gulf of Guinea countries, and is a source of conflict for developing countries and sub-Saharan countries in particular [47,48]. This curse is not unique to the Gulf of Guinea countries; it has been observed in Malaysia [4]. In order to overcome this situation, it would be interesting to optimise the management of its oil resources by, for example, directing the oil rent towards wealth-creating economic sectors.

Cameroonian crude prices are set according to Dated Brent from the North Sea and listed on the London Stock Exchange [49]. This suggests that Cameroon does not set the price of its crude. The lack of control over this important factor exposes the country to uncertainty about production and the value of the oil rent. Thus, when the price of crude rises or experiences a positive long-term shock, it allows production to increase [1,39] and oil rents can increase dramatically [5]. On the other hand, a fall in the price of crude can lead to a fall in crude production and consequently to a fall in oil rent as it is influenced by the price, cost and quantity of production [4].

However, there is a unidirectional causality from corruption to crude production. [44] and [43] respectively, indicate that an increase in corruption is associated with significantly lower levels of crude production and that corruption can reinforce lower than potential crude production. It is easy to see that if crude production is negatively affected by oil rent, this would certainly be due to the pervasiveness of corruption in the sector [50]. Moreover, in 2021, the world corruption ranking identifies Cameroon in 144^ème^ place out of 180 countries identified with a survey listing that 72% of people think that corruption has increased over the last twelve months [51].

The bidirectional causality found between the development index and corruption partly reflects the role that the HDI plays on corruption and crude production. Indeed, the HDI is reported in the literature as a useful element on which countries or states can build to significantly reduce corruption. The work of [52] illustrates this. That said, the HDI can be a catalyst for reducing the perceived negative effects of corruption on crude production. And if corruption is reduced, production can benefit the oil rent. Moreover, in 2019, Cameroon is better ranked in terms of the HDI than the average for sub-Saharan Africa, at 0.56 and 0.547 respectively [53].

Finally, the unidirectional causality from the HDI to crude production reflects the major role that the HDI plays in crude production. Indeed, the HDI, through the development of human capital in the field of health, education and training, makes it possible to have a qualified workforce to improve productivity [42]. Thus, it can be seen that the HDI is an important factor that can act in the production of crude and help reverse the curse situation. In addition, the HDI has a positive and significant impact on oil rent in the long-term. This result reflects the importance of developing human capital to create extraordinary benefits in the oil sector in Cameroon. By the way, [6] recommends using human development to offset the negative effects of oil rent on financial development.

## Conclusions

This study examined the relationship between oil rent and crude oil production in Cameroon over the period 1977 to 2019. It used ARDL modelling and the Granger causality test in the Toda–Yamamoto sense to do so. The results of this study show that a 1% increase in crude oil production leads to a 0.81% decrease in oil rent and that there is a bidirectional causality between oil rent and crude oil production. They also indicate that production is the transmission node of the effects of crude oil price, HDI and corruption on oil rent. These results could mean that Cameroon is under the oil curse. Since the production of crude oil has perverse effects on the oil rent, the latter could therefore compromise the exploitation of crude oil in Cameroon. For its emergence, Cameroon relies on oil revenues to not only balance its budget but also to contribute to its development. Variables such as the price of crude oil, HDI and corruption could therefore play a key role in mitigating this oil curse. In light of these findings, Cameroonian decision-makers should put in place satisfactory mechanisms to prevent fluctuations in crude oil prices, increase an optimal social policy to fight corruption in the oil sector, and finally, truly direct the oil rent towards the country's economic circuit.

## CRediT authorship contribution statement

**Marcel Rodrigue Ewodo-Amougou:** Conceptualization, Methodology, Software, Writing – original draft. **Flavian Emmanuel Sapnken:** Validation, Data curation, Visualization, Investigation. **Inoussah Moungnutou Mfetoum:** Software, Writing – review & editing. **Jean Gaston Tamba:** Supervision, Validation, Writing – review & editing.

## Declaration of Competing Interest

The authors declare that they have no known competing financial interests or personal relationships that could have appeared to influence the work reported in this paper.

## Data Availability

Data will be made available on request.
